# Flavonoids: Antioxidant Powerhouses and Their Role in Nanomedicine

**DOI:** 10.3390/antiox13080922

**Published:** 2024-07-29

**Authors:** Mehak Zahra, Heidi Abrahamse, Blassan P. George

**Affiliations:** Laser Research Centre, Faculty of Health Sciences, University of Johannesburg, P.O. Box 1711, Doornfontein 2028, South Africa; mehakzahra.zm@gmail.com (M.Z.); habrahamse@uj.ac.za (H.A.)

**Keywords:** antioxidant, anti-inflammatory, flavonoids, natural products, nanoparticles, South Africa

## Abstract

This study emphasizes the critical role of antioxidants in protecting human health by counteracting the detrimental effects of oxidative stress induced by free radicals. Antioxidants—found in various forms such as vitamins, minerals, and the phytochemicals abundant in fruits and vegetables—neutralize free radicals by stabilizing them through electron donation. Specifically, flavonoid compounds are highlighted as robust defenders, addressing oxidative stress and inflammation to avert chronic illnesses like cancer, cardiovascular diseases, and neurodegenerative diseases. This research explores the bioactive potential of flavonoids, shedding light on their role not only in safeguarding health, but also in managing conditions such as diabetes, cancer, cardiovascular diseases, and neurodegenerative diseases. This review highlights the novel integration of South African-origin flavonoids with nanotechnology, presenting a cutting-edge strategy to improve drug delivery and therapeutic outcomes. This interdisciplinary approach, blending traditional wisdom with contemporary techniques, propels the exploration of flavonoid-mediated nanoparticles toward groundbreaking pharmaceutical applications, promising revolutionary advancements in healthcare. This collaborative synergy between traditional knowledge and modern science not only contributes to human health, but also underscores a significant step toward sustainable and impactful biomedical innovations, aligning with principles of environmental conservation.

## 1. Introduction

Antioxidants are a class of compounds that play a crucial role in maintaining human health by protecting the body from oxidative stress and the damage caused by harmful molecules known as free radicals. These molecules are highly reactive and can lead to cellular damage, DNA mutations, and various diseases if not properly controlled [1]. Antioxidants work by neutralizing free radicals, preventing them from causing harm to cells and tissues. They do this by donating electrons to stabilize these highly reactive molecules, thereby reducing their potential for damage [2]. There are numerous types of antioxidants, including vitamins (such as vitamin C and vitamin E), minerals (like selenium and zinc), and the phytochemicals found in fruits, vegetables, and other plant-based foods. Each type of antioxidant has its unique mechanism of action and may be more effective against specific types of free radicals [3]. Consuming antioxidant-rich foods is associated with a lowered risk of chronic diseases, such as heart disease, cancer, and neurodegenerative disorders [4].

Antioxidants are believed to help combat inflammation, support the immune system, and slow the aging process by reducing oxidative stress [4]. However, it is important to maintain a balanced intake of antioxidants as excessive supplementation can have negative effects and potentially disrupt the delicate balance of oxidative reactions within the body. A well-rounded diet that includes a variety of fruits, vegetables, and whole grains is typically the best way to ensure a healthy intake of antioxidants [5]. Antioxidants are essential components of a healthy diet and play a significant role in protecting the body from the damaging effects of oxidative stress and free radicals, ultimately promoting overall human health and well-being [3].

The Phyllanthaceae family, a large group of flowering plants with approximately 1301 species, includes the genus Phyllanthus. This genus is widely distributed across the tropical and subtropical regions of Africa, America, Asia, and Australia [6,7]. Historically, Phyllanthus *Cicca* and *P. Kirganelia* have been the most significant species within the Phyllanthaceae family for treating various human ailments [8]. Phyllanthus plants are rich in phytochemical components important in pharmacology, including terpenoids, alkaloids, and polyphenolic substances such as phenolic acids, flavonoids, coumarins, lignins, stilbenes, and anthocyanins [9]. In South African flora, many plant species are rich sources of flavonoids, and traditional medicine systems have utilized these bioactive compounds for centuries [10]. Flavonoids are well known for their potent antioxidant properties, which play a crucial role in protecting cells and tissues from oxidative stress. This is essential for maintaining overall health and preventing various chronic diseases [11]. A high consumption of fruits and vegetables has been shown to correlate with a reduced incidence and mortality rate of various degenerative diseases, including cancer, cardiovascular disease, and immune dysfunction, according to multiple human cohort and case–control studies [12,13,14]. Alongside the vitamins and minerals found in fruits and vegetables, phytochemicals such as flavonoids and other phenolics may contribute to these protective benefits. Estimating flavonoid intake is an essential step toward understanding their protective effects against chronic diseases [15]. Although flavonoids are significant dietary antioxidants, the limited data on their comprehensive food composition has hindered the assessment of dietary intake in populations. A study by Chun et al. indicated that the highest daily mean intake of flavonoids came from tea (157 mg), citrus fruit juices (8 mg), wine (4 mg), and citrus fruits (3 mg) [16]. The efficacy of flavonoids is highly dependent on their bioavailability and metabolism. The concentration needed to achieve therapeutic effects can vary based on the form of flavonoid, how it is consumed, and individual differences in metabolism [11]. Flavonoid supplements are associated with numerous health benefits, including antioxidant, anti-inflammatory, and anticancer properties [17]. Regular consumption of flavonoid-rich supplements can help in reducing the risk of chronic diseases such as cardiovascular diseases, diabetes, and certain types of cancer [18]. Common flavonoid supplements include quercetin, EGCG, and genistein, which are available in various forms such as capsules, tablets, and powders. These supplements are often combined with other vitamins and minerals to enhance their health effects [19]. Kaempferol, also known as 3,4′,5,7-tetrahydroxyflavone, is a flavonol with hydroxyl groups at positions 3, 5, 7, and 4′ [20]. It can be found in various sources such as broccoli, cabbage, gooseberries, grapes, kale, strawberries, tomatoes, citrus fruits, brussels sprouts, and several medicinal plants. Kaempferol has demonstrated strong binding affinities with EGFR, surpassing those of the native ligand and even gefitinib [21]. Inflammation is a key factor in the development of many diseases, including cardiovascular diseases and cancer. Flavonoids have demonstrated anti-inflammatory properties that can help mitigate the inflammatory processes in the body [20]. The antioxidant and anti-inflammatory activities of flavonoids have also been associated with a reduced risk of cancer development. These compounds may inhibit the growth of cancer cells and promote apoptosis, making them a potential component in cancer prevention [22]. Flavonoids have also been linked to improving cardiovascular health due to their ability to reduce oxidative stress and inflammation in blood vessels. This can help prevent atherosclerosis and reduce the risk of heart diseases [23]. They have also shown promise in protecting the brain from oxidative damage and inflammation, potentially reducing the risk of neurodegenerative diseases such as Alzheimer’s and Parkinson’s [24]. Many studies have indicated that flavonoids may help manage diabetes by improving insulin sensitivity and reducing inflammation, making them valuable in the management of this chronic condition [25].

The study of flavonoids’ bioactive properties may lead to the development of new pharmaceuticals or nutraceuticals for various health conditions, offering alternatives or complementary approaches to traditional therapies. By elucidating and harnessing the antioxidant and anti-inflammatory potential of flavonoids, researchers can contribute to the development of strategies and products that have a positive impact on human health and well-being [26]. Nanomedicine employs biocompatible nanomaterials for therapeutic interventions in the treatment of diverse diseases. Flavonoids derived from plant materials serve a dual role as reducing and electrostatic stabilizing agents, facilitating the green synthesis of metal nanomaterials. These synthesized nanomaterials demonstrate effective applications in treating cancer cells and combating pathogenic microbes [27]. Flavonoid-loaded nanoparticles have demonstrated effectiveness in preventing bacterial cell damage in oxidizing environments. The prolonged antioxidant effect observed in the oxidizing medium can be attributed to the gradual release of flavonoids from the nanoparticles. This is in contrast to free flavonoids, which are largely depleted in the initial phase due to their rapid consumption [28]. Encapsulating flavonoids in nanoparticles enhances their effects on cytokine production in vitro and in vivo. This is due to improved cellular uptake as nanoparticles, with their small size and surface traits, facilitate membrane interaction, resulting in higher intracellular flavonoid concentrations. Additionally, encapsulation protects flavonoids, increasing their stability and bioavailability, thereby preventing degradation and improving absorption and circulation [29]. Understanding the interaction between cell membranes and nanoparticles is crucial for evaluating the efficiency of flavonoid-loaded nanoparticles. Hydrophobic interactions between polyphenols and liposome membranes, for instance, enhance the encapsulation and delivery of polyphenols. This interaction facilitates efficient loading into liposomes, protecting the polyphenols from degradation and enhancing their stability during storage and transport [29].

This review focuses on novel studies about the treatment potential of flavonoids and their fusion with nanotechnology, presenting a cutting-edge approach that could revolutionize drug delivery and improve therapeutic outcomes. Integrating traditional wisdom with contemporary techniques not only enriches our comprehension of these compounds, but it also facilitates their efficient utilization in nanomedicine.

## 2. South African Flora: A Rich Source of Flavonoids

The geographical distribution of key plants in South Africa is remarkably diverse, and this diversity extends to the presence of various flavonoids within these plants (Table 1). Among these botanical treasures, *Rooibos* stands out as a beloved South African plant, thriving in the Western and Northern Cape regions. *Rooibos* is renowned for its rich flavonoid composition, and it is particularly characterized by flavonols like quercetin and aspalathin [30]. Indigenous South African communities have traditionally used *rooibos*, such as the Khoi and San, for centuries, as shown in Table 1. It has been consumed as an herbal infusion for its refreshing taste and potential health benefits [31]. Rooibos tea is abundant in flavonoids like aspalathin and nothofagin, which are known for their potent antioxidant properties. These flavonoids help neutralize free radicals, reducing oxidative stress and preventing cellular damage [31]. Research indicates that the flavonoids in rooibos tea can enhance lipid profiles and lower blood pressure, contributing to cardiovascular health. Regular consumption of rooibos tea may aid in the prevention of cardiovascular diseases. Aspalathin, a distinctive flavonoid in rooibos, has shown promise in regulating blood sugar levels by improving insulin sensitivity and reducing fasting glucose levels, making it a beneficial dietary supplement for diabetes management [32]. *Cyclopia intermedia* is indigenous to South Africa, and it particularly thrives in the fynbos biome spanning the Western and Eastern Cape provinces. This area boasts diverse flora and rich biodiversity, making it a prime habitat for the *Cyclopia* species, which are widely cultivated for the production of honeybush tea [33]. This plant is also utilized to create a sweet herbal infusion known for its therapeutic effects, including soothing coughs and easing respiratory issues like tuberculosis, pneumonia, and catarrh [33]. In the arid landscapes of South Africa, another notable indigenous species, *Hoodia gordonii*, finds its habitat. While *Hoodia gordonii* is primarily recognized for its appetite-suppressing properties, it also possesses flavonoids such as quercetin and kaempferol [34]. *Hoodia gordonii* has a history of use by San Bushmen in South Africa as an appetite suppressant during long hunting trips [35]. *Hoodia* contains flavonoid compounds that might contribute to weight management by reducing caloric intake and supporting weight loss. Although mainly recognized for its appetite suppression, it is also thought to boost energy levels, though scientific evidence for this effect is limited [36]. The South African region is also home to various cycad species, and these plants often harbor flavonoids among their diverse phytochemical constituents. Traditional uses of cycads among South African indigenous communities include the consumption of certain species as a starchy food source, although this practice has raised conservation concerns [37]. Notably, *Pelargonium sidoides*, commonly referred to as *Umckaloabo*, is a medicinal plant native to South Africa. It is celebrated for its flavonoid-rich composition, particularly in the form of quercetin derivatives [38]. *Pelargonium sidoides* has been traditionally used by South African communities, including the Zulu and Xhosa, for its medicinal properties in the treatment of respiratory infections [38]. Additionally, the South African region is host to *Sceletium tortuosum*, a plant with a historical and cultural significance. *Sceletium tortuosum*, which is native to this region, contains mesembrine alkaloids, which are a distinctive type of flavonoid [39]. *Sceletium tortuosum* has a long history of traditional use among indigenous South African communities, such as the San and Khoi, as a mood-enhancing and stress-reducing herb [40]. These diverse plant species and their flavonoid-rich compositions underscore the unique botanical wealth of South Africa and its importance in the study of flavonoids within a variety of ecological and pharmacological contexts.

## 3. Antioxidant Potential of Flavonoids

**Figure 1:**
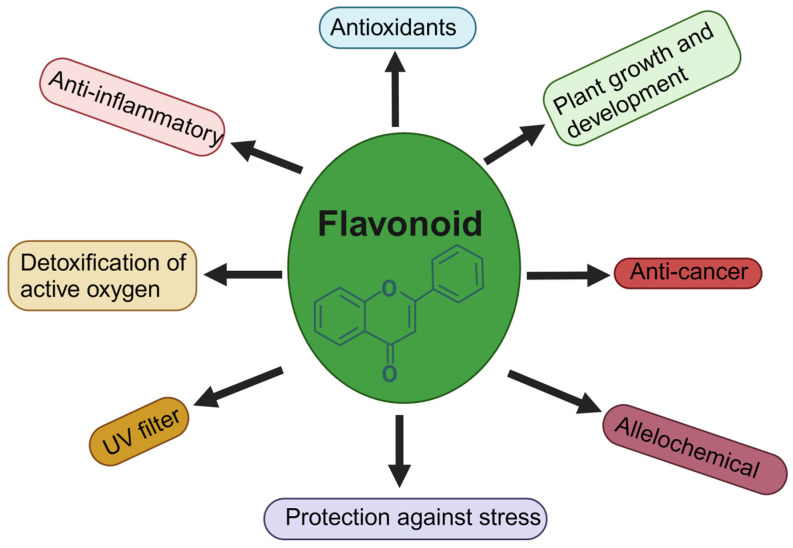
The role and functional significance of flavonoids: an illustrated overview.

Antioxidants, such as flavonoids, in preventing oxidative stress-related diseases, are supported by various studies that highlight their impact on mitigating cellular damage and disease development. Free radicals, or ROS, are inherently unstable molecules generated by living organisms as part of routine cellular metabolism. When there is a tilt in the equilibrium between oxidants and antioxidants in favor of oxidants, this condition is referred to as oxidative stress [41]. Flavonoids are potent antioxidants with multiple mechanisms that contribute to their effectiveness in neutralizing free radicals and preventing oxidative damage in the body. Flavonoids act as free radical scavengers, neutralizing reactive oxygen species (ROS) through their ability to donate hydrogen atoms or electrons [42]. In cardiovascular diseases, flavonoids help to improve endothelial function and reduce inflammation by scavenging free radicals [43]. Some flavonoids exhibit metal chelation properties, binding to transition metals such as iron and copper, which can otherwise catalyze the generation of free radicals. This chelation inhibits metal-induced oxidative stress [44]. Flavonoids possess the capability to hinder the activity of enzymes that generate reactive species like xanthine oxidase and NADPH oxidase, thereby diminishing the production of ROS [45]. Additionally, flavonoids have the ability to restore other antioxidants such as vitamin C and vitamin E, thus bolstering the body’s overall antioxidant defense system [46]. In vitro studies focusing on South African flavonoids have unveiled their robust antioxidant capabilities through various experimental setups and assessments. Research conducted by Oyedemi et al. examined the antioxidant potential of South African plant extracts rich in flavonoids using 1,1-diphenyl-2-picrylhydrazyl (DPPH) radical scavenging assays, demonstrating significant radical scavenging activity [47]. Studies by Firuzi et al. further explored the antioxidant activity of South African flavonoids through ferric-reducing antioxidant power (FRAP) assays, indicating their capacity to reduce ferric ions, a marker of antioxidant potential [48]. In an investigation by Aderogba et al., South African plant extracts, particularly flavonoid-rich ones, were tested for their total antioxidant capacity, demonstrating significant antioxidant properties through various in vitro assays [49]. The fermented leaves and stems of *Cyclopia intermedia* are utilized to create Honeybush tea, a traditional herbal infusion native to South Africa. This plant is also processed into a sweet herbal drink known for its therapeutic properties, including soothing coughs and easing respiratory conditions like tuberculosis, pneumonia, and catarrh. Honeybush tea is reputed to be low in tannins and being caffeine-free while also containing a range of antioxidants [33]. Marnewick et al. explored the antioxidant characteristics of several South African herbal teas, notably *rooibos* (*Aspalathus linearis*) and honeybush (*Cyclopia* spp.), both of which contain distinctive flavonoids like aspalathin and mangiferin [31]. Another study indicated that honeybush tea, brewed from *Cyclopia intermedia*, contains a substantial quantity of polyphenols, which contribute to its various physiological benefits. Traditionally used as a medicinal drink, honeybush tea is now gaining recognition for its health-promoting properties, including its antioxidant activity and low caffeine content [50]. Several roles and the functional significance of flavonoids are illustrated in Figure 1.

### 3.1. Anti-Inflammatory and Anti-Oxidative Flavonoids

**Figure 2:**
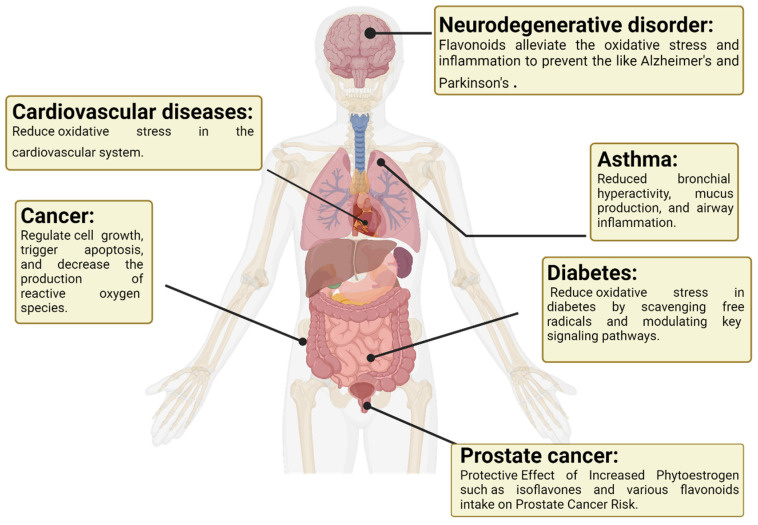
Flavonoids prevent oxidative stress-related diseases.

Oxidative stress stands as the primary underlying factor for conditions like cancer, diabetes, arthritis, rheumatoid arthritis, neurodegenerative disorders, hypertension, atherosclerosis, and chronic inflammatory ailments [51]. Research by Ciumarnean et al. suggested a protective association between flavonoid intake and a reduced risk of cardiovascular diseases, attributing this effect to the antioxidant properties of flavonoids, particularly their ability to reduce oxidative stress in the cardiovascular system [52]. As shown in Figure 2, in the development of cardiovascular disease, a crucial inflammatory process occurs. Various research studies have connected inflammatory and immune responses to the vascular damage linked with atherosclerosis [53]. Oxidative stress triggers the elevation of enzymes like cyclooxygenase (COX) and lipoxygenase (LPO), which contribute to the release of chemokine factors such as interleukins [54,55]. Notably, certain polyphenols, especially quercetin, have demonstrated the ability to inhibit COX and LPO enzymes, potentially curbing this inflammatory cascade [56]. Polyphenols exhibit anti-inflammatory characteristics and can regulate inflammatory mediators in individuals at a heightened risk of cardiac disease (Figure 2) [57]. Flavonoids have become a focal point for specialists due to their diverse potential benefits. Research on these compounds is intricate and time-consuming because of their varied molecular structures. However, several studies have proposed that dietary polyphenols could be advantageous as a supplementary approach in preventing and treating chronic inflammatory conditions [57,58]. Studies, such as those by Meng-zhen, demonstrate the potential of flavonoids in mitigating oxidative stress and inflammation in neurodegenerative diseases, thereby suggesting a role in preventing conditions like Alzheimer’s and Parkinson’s [59]. Flavonoids have shown promise in cancer prevention due to their antioxidative and anti-inflammatory properties. Flavonoids function as signaling molecules that regulate cell growth, trigger apoptosis, and decrease the production of reactive oxygen species. These capabilities offer potential alternative strategies for both treating and preventing cancer [60]. Quercetin, a naturally occurring flavonoid present in numerous fruits and vegetables, has demonstrated diverse biological effects in experimental models. Among its observed effects is the alleviation of key symptoms associated with asthma, including bronchial hyperactivity, mucus production, and airway inflammation (Figure 2) [61]. The impact of flavonoids on oxidative stress and diabetes has been explored in previous studies, indicating that these antioxidants can help reduce oxidative stress in diabetes by scavenging free radicals and modulating key signaling pathways [62]. The World Health Organization identified cardiovascular disease (CVD) as the leading cause of death worldwide in 2019. Previous research has indicated differences in the composition of the gut microbiome between individuals with and without CVD, and it has been suggested that flavonoids may reduce the risk of heart disease [63].

The body initiates inflammation as a normal biological response to tissue injury, microbial pathogen infection, and chemical irritation. This process involves the migration of immune cells from blood vessels, releasing mediators at the site of damage. Following this, inflammatory cells are recruited, and there is a release of ROS, reactive nitrogen species (RNS), and proinflammatory cytokines to eliminate foreign pathogens and aid in tissue repair. Typically, normal inflammation is a quick and self-limiting process, but if resolution is aberrant and inflammation persists, it can contribute to the development of various chronic disorders [64]. Persistent inflammation and oxidative stress play crucial roles in the development of obesity, cancer, and neurodegenerative diseases. Flavonoids are gaining recognition as promising therapeutic agents for these conditions, given their anti-inflammatory and antioxidant properties [60]. Several flavonoids, including hesperidin, apigenin, luteolin, and quercetin, have been documented for their anti-inflammatory and analgesic properties. These flavonoids may specifically impact the function of enzyme systems that play a crucial role in the initiation of inflammatory processes, particularly tyrosine and serine-threonine protein kinases [65]. Flavonoids exert their kinase inhibition by competitively binding with ATP at the catalytic sites of these enzymes. The targeted enzymes play crucial roles in signal transduction and cell activation processes, particularly within the immune system’s cells. Research indicates that flavonoids have the capability to inhibit the expression of isoforms of inducible nitric oxide synthase, cyclooxygenase, and lipoxygenase. These enzymes are responsible for generating significant amounts of nitric oxide, prostanoids, leukotrienes, and other inflammatory mediators, including cytokines, chemokines, and adhesion molecules [66]. Elisha et al. [67] conducted an in vitro study on acetone leaf extracts from nine selected plants, revealing significant antioxidant, anti-inflammatory, and anti-arthritic properties. This research aligns with traditional assertions regarding the efficacy of these South African medicinal plants in addressing conditions such as arthritis, infections, rheumatism, and inflammation. The identified plants exhibit potential for becoming therapeutic agents in the treatment of inflammation and other autoimmune disorders.

**Figure 3:**
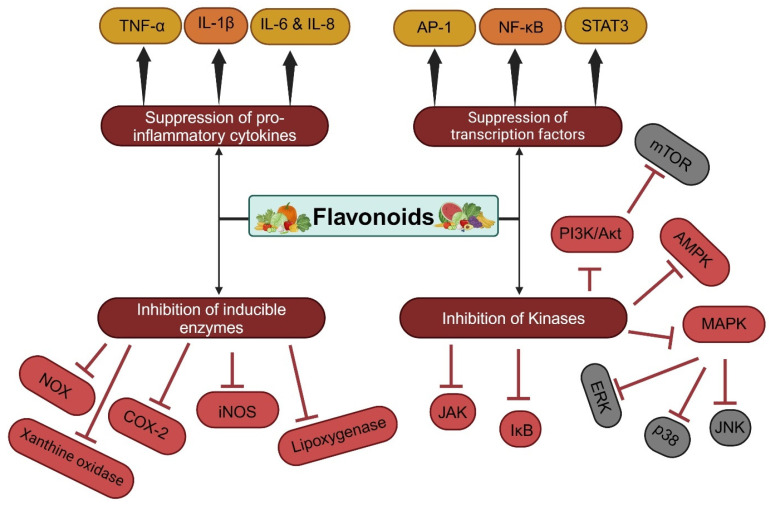
The targets of flavonoids in inflammatory processes.

Natural compounds, including flavonoids, exhibit neuroprotective capabilities, likely attributed to their capacity to regulate the inflammatory responses implicated in neurodegenerative diseases [68]. Numerous studies have proposed a potential neuroprotective and anti-inflammatory function of plant extracts that are rich in flavonoids. For instance, the anti-inflammatory potential of honey flavonoid extract (HFE) was evaluated using N13 microglia cells stimulated by lipopolysaccharide. In this model, HFE (at flavonoid concentrations of 0.5 and 1 mg/mL) significantly reduced the generation of pro-inflammatory mediators, specifically inhibiting the expression of inducible nitric oxide synthase (iNOS) mRNA and protein levels. Additionally, HFE demonstrated a notable suppression in the production of both tumor necrosis factor-α (TNF-α) and interleukin-1β (IL-1β) [69]. As shown in Figure 3, flavonoids have been demonstrated to exhibit anti-inflammatory effects by modulating immune cell function, reducing chemokine and COX-2 expression, suppressing cytokine release, and inhibiting pro-inflammatory transcription factors such as PI3K/Akt and IKK/JNK [42,70]. A recent study highlighted the potential of encapsulated flavonoids in modulating cytokine activity in various in vitro and in vivo models. Encapsulated flavonoids can influence cytokine activity differently, depending on the flavonoid type, cell line, concentration, and treatment duration. Some studies have shown the downregulation of cytokines like IL-1β, IL-6, TNF-α, and IL-8, while others have noted the upregulation of IL-10. Encapsulated flavonoids often have a more significant impact on cytokine activity compared to their non-encapsulated counterparts [29]. Flavonoids can modulate various cell signaling pathways involved in oxidative stress, inflammation, and apoptosis. These pathways can be differentially regulated in cancer and non-cancerous conditions. For example, in non-cancerous cells, flavonoids can activate the Nrf2 pathway, leading to the upregulation of antioxidant defenses. In cancer cells, flavonoids might inhibit the NF-κB pathway, reducing cell survival and proliferation [71]. The therapeutic effects of flavonoids, particularly their modulation of ROS, differ between cancerous and non-cancerous conditions due to variations in cellular redox states, metabolic requirements, and signaling pathways [72]. In cancerous cells, the redox balance is often disrupted, leading to elevated levels of ROS [2,73]. These elevated ROS levels can promote cancer cell proliferation, survival, and metastasis through various signaling pathways, such as the MAPK, PI3K/Akt, and NF-κB pathways [74]. Flavonoids can modulate these pathways by reducing ROS levels, thereby inhibiting cancer cell growth and inducing apoptosis [75]. For example, quercetin has been shown to induce apoptosis in cancer cells by modulating the PI3K/Akt pathway and reducing ROS levels [76]. On the other hand, in non-cancerous cells, flavonoids can help maintain redox homeostasis and protect against oxidative stress-induced damage. This protective effect is particularly beneficial in conditions such as cardiovascular diseases, neurodegenerative disorders, and aging, where oxidative stress plays a key role [77]. Flavonoids like kaempferol and epicatechin have been found to enhance antioxidant defenses and reduce ROS levels in non-cancerous cells, thereby preventing cellular damage and promoting overall health [75]. Despite the promising therapeutic potential, several challenges need addressing for the effective utilization of South African flavonoids in clinical settings. The standardization of extraction processes, identification of optimal dosages, and clarification of potential interactions with conventional medications are crucial areas that require attention. Future research should focus on unraveling the specific mechanisms of action of different South African flavonoids, considering their interactions within complex cellular pathways. Moreover, the exploration of synergistic effects among flavonoids and with other bioactive compounds present in South African plants is a promising avenue for future investigations.

### 3.2. Anti-Cancer Effects of Flavonoids

Flavonoids have been consistently shown in various studies to possess the capacity to neutralize free radicals, modulate cellular metabolism, and mitigate the risk of diseases associated with oxidative stress [78]. Numerous studies have suggested that a variety of flavonoids demonstrate anticancer properties. Despite this, the precise molecular mechanisms underlying these effects remain incompletely understood. Cancer originates from internal factors such as oxidative stress, hypoxia, genetic mutations, and deficient apoptotic function [70]. Studies by Kopustinskiene et al. [75] have emphasized the role of flavonoids in reducing oxidative stress and preventing DNA damage, which are critical factors in cancer development. The role of oxidative stress in initiating cancer is well established, and potent antioxidants have the potential to impede the progression of carcinogenesis. The effectiveness of an antioxidant compound to act as an anticancer agent relies on its ability to neutralize oxygen radicals and act as an inhibitor. Diets abundant in radical scavengers have the capacity to reduce the cancer-promoting effects of certain radicals [79]. Among the most common genetic abnormalities in human cancers are mutations in the p53 gene. Inhibiting the expression of p53 may halt the progression of cancer cells in the G2-M phase of the cell cycle. In human breast cancer cell lines, flavonoids have been shown to effectively decrease the expression of the mutant p53 protein, bringing it down to nearly undetectable levels [80]. External factors contributing to its development include exposure to stress, pollution, smoking, radiation, and ultraviolet rays [81]. Cancer cells exhibit distinctive traits, including modified metabolism, disrupted cell cycles, frequent genetic mutations, resilience against immune responses, persistent inflammation, development of metastases, and stimulation of angiogenesis [82]. External factors contributing to its development include exposure to stress, pollution, smoking, radiation, and ultraviolet rays [81,82]. Flavonoids display a diverse range of anticancer properties, influencing the activities of enzymes involved in scavenging ROS. They contribute to cell cycle arrest, promote apoptosis and autophagy, and inhibit the proliferation and invasiveness of cancer cells [75,83,84]. When the balance of pro-oxidant activities and the antioxidant defense within cells is disrupted, there is a rise in ROS production, resulting in the accumulation of free radicals [83]. ROS primarily originate in the mitochondrial electron transport chain as byproducts of the oxidative phosphorylation in cells [85]. The increased levels of ROS contribute to oxidative stress, which plays a role in the onset of inflammatory processes associated with various degenerative diseases and cancer. Flavonoids exhibit a dual role in maintaining ROS homeostasis: they function as antioxidants under normal conditions and serve as potent pro-oxidants in cancer cells, activating apoptotic pathways [86,87]. Flavonoids exhibit direct ROS-scavenging capabilities and can chelate metal ions by stabilizing free radicals through the presence of phenolic hydroxyl groups [88,89]. The indirect antioxidant effects of flavonoids are associated with the activation of antioxidant enzymes, as well as the inhibition of pro-oxidant enzymes and phase II detoxification enzymes. Both antioxidant and pro-oxidant activities contribute to the anticancer effects of flavonoids [2]. A recent in vivo study suggested that a specific polyphenolic compound found in green tea, which consists mainly of flavanol and proanthocyanidin components, effectively prevents the formation of skin tumors in mice. Additionally, it showed that, for the first time, certain fractions that are extracted from rooibos and honeybush, using a method involving ethyl acetate, also protect against the tumor promotion induced by TPA (a chemical compound) in mouse skin. Their findings indicate a significant reduction in the number of tumors, a decrease in their size, and a delay in their formation [90]. Another study suggested that rooibos and honeybush extracts, like green tea extracts, disrupt cell growth by affecting mitochondrial function, causing mitochondrial membrane depolarization. They inhibit cell proliferation at lower concentrations and induce apoptosis at higher concentrations, targeting precancerous cells. The anticancer effects of green tea and rooibos extracts primarily involve monomeric flavonoids, while honeybush extracts, rich in polymeric proanthocyanidins, alter cell growth parameters and often exhibit protective effects with their monomeric polyphenols. These herbal teas’ ability to prevent cancer is influenced by specific interactions between polyphenols and cells, which vary between extracts and contribute to their unique anticancer properties when compared to green tea [91]. Genistein, an isoflavone, facilitates the arrest of breast cancer MDA-MB-231 and MCF-7 cells at the G2/M phase, leading to a subsequent apoptosis dependent on ROS [92]. As shown in Table 2, daidzein induces apoptosis in MCF-7 breast cancer cells by generating ROS [93]. Hesperetin, a flavanone, triggers apoptosis in gall bladder carcinoma [94], esophageal cancer [95], hepatocellular carcinoma [96], and human breast carcinoma MCF-7 cells [97]. This effect is achieved by activating the mitochondrial apoptotic pathway through increased ROS production. Naringenin, another flavanone, exhibits anticancer effects on choriocarcinoma JAR and JEG 3 cell lines by inducing ROS generation and activating signaling pathways [98]. Additionally, it initiates an apoptotic cascade in human epidermoid carcinoma A431 cells [99]. In prostate cancer PC3 and LNCaP cell lines, naringenin suppresses proliferation and migration, induces apoptosis, and generates ROS (Table 2) [100]. An increased intake of phytoestrogens, such as isoflavones and various flavonoids, demonstrates a protective effect against the risk of prostate cancer [101]. Moreover, naringenin has demonstrated the ability to decrease the generation of ROS while augmenting the activity of superoxide dismutase, catalase, and glutathione in chronic diseases and cancer [102]. The pro-oxidant properties of cocoa catechins and procyanidins have been observed to induce apoptotic morphological changes and DNA damage, leading to apoptosis in epithelial the ovarian cancer cells OAW42 and OVCAR3 [103]. Flavonoids appear to be significant anti-cancer agents by targeting receptor tyrosine kinases (RTKs) and influencing their downstream signaling pathways, such as MAPK, PI3K/Akt, and JAK/STAT [104]. The development of various cancers may be linked to the activation of specific proto-oncogenes, such as EGFR. EGFR is widely distributed in mammalian epithelial cells and binds to epidermal growth factor (EGF) or tumor necrosis factor (TNF) [105,106]. A study by Chen et al. found that quercetin reduces cervical cancer cell viability, promotes G2/M phase cell cycle arrest, induces apoptosis, and inhibits cell migration and invasion. The activation of the EGFR and ERK pathways has been observed, with both kinases markedly activated by quercetin. Inhibition of these pathways using afatinib (EGFR inhibitor) and U0126 (ERK inhibitor) enhances apoptosis and cell cycle arrest, suggesting that EGFR and ERK activation may counteract the anticancer effects of quercetin [107]. In HepG2 cells, cocoa polyphenolic extract activates the ERK1/2 pathway, enhancing the activities of glutathione peroxidase and reductase [108]. Additionally, cocoa catechins and procyanidins provides protection to Caco2 cells against induced oxidative stress, reducing ROS production and preventing cellular death [109]. The antioxidant properties of cocoa flavanols have demonstrated beneficial effects in safeguarding against colon cancer [110]. Recent studies have indicated that quercetin reduces the proliferation of hepatocellular carcinoma HepG2 cells by decreasing intracellular ROS levels [111]. Moreover, it has been shown to increase ROS production and the number of apoptotic cells in human gastric cancer AGS and human breast cancer MCF-7 cells [112]. Kaempferol, a flavonol, impedes the growth of bladder cancer EJ cells by inducing apoptosis and causing S phase arrest through the modulation of ROS levels [113]. In colorectal cancer cell lines (HCT116, HCT15, and SW480), kaempferol activates caspases through ROS generation, leading to apoptosis [114]. Additionally, kaempferol exhibits cytotoxic effects on rat hepatocellular carcinoma cells by targeting mitochondria through ROS mediation [115]. The anticancer properties of flavones apigenin and luteolin in ovarian cancer cell lines (A2780, OVCAR-3, and SKOV-3) have been associated with alterations in ROS signaling and the promotion of apoptosis [116]. Apigenin also induces apoptosis in human cervical cancer-derived cell lines, including HeLa, SiHa, CaSki, and C33A, through increased ROS generation and the initiation of mitochondrial apoptotic pathways [117]. Flavone chrysin increases ROS and lipid peroxidation levels, leading to the death of choriocarcinoma (JAR and JEG3), bladder, and ovarian cancer (ES2 and OV90) cells [118,119]. The antioxidant activity of flavonoids has been explored in human studies, revealing a correlation between serum total antioxidant capacity and dietary anthocyanin consumption [120]. Cyanidin induces cell death in DU145 and LnCap human prostatic cancer cells through ROS modulation [121]. Cyanidin and delphinidin accelerates cellular ROS accumulation, suppresses glutathione reductase, and depletes glutathione, resulting in cytotoxicity in metastatic colorectal cancer cells (LoVo and LoVo/ADR) [122]. Quercetin and daidzin are two natural compounds currently receiving significant attention for their anticancer properties [123]. Quercetin, a flavonoid primarily found in vegetables and fruits such as capers, lovage, dill, cilantro, and onions, has been reported to inhibit HeLa cells by blocking the phosphatidylinositol 3-kinase (PI3K)-Akt/PKB (protein kinase B) pathway. It also promotes apoptosis through the activation of the intrinsic apoptotic pathway, which includes the upregulation of Bax, Bad, Bid, caspase-9, and caspase-3, as well as the downregulation of Bcl-2 and Bcl-xL. Additionally, quercetin triggers cytochrome c release and inhibits NF-κB, PKC-δ, and ERK1/2 while activating AMPK and reducing uPA/uPAR, MMP-9, and MMP-2 to hinder cell migration and invasion [124,125,126]. Daidzin, a glycoside form of the flavonoid daidzein and found in soy isoflavones, has been noted for its anticancer effects in the early stages of prostate cancer development and for reducing the risk of postmenopausal breast cancer [127]. It is also known to inhibit telomerase activity by forming hydrogen bonds with the base of the G-quadruplex [128,129]. A recent study by Zubair et al. revealed that daidzin is more cytotoxic and selective on T47D and HeLa cell lines than quercetin, likely due to its glucose units forming hydrogen bonds with specific amino acid residues. Docking results have supported this, showing lower binding energy for daidzin compared to quercetin [130].

**Figure 4:**
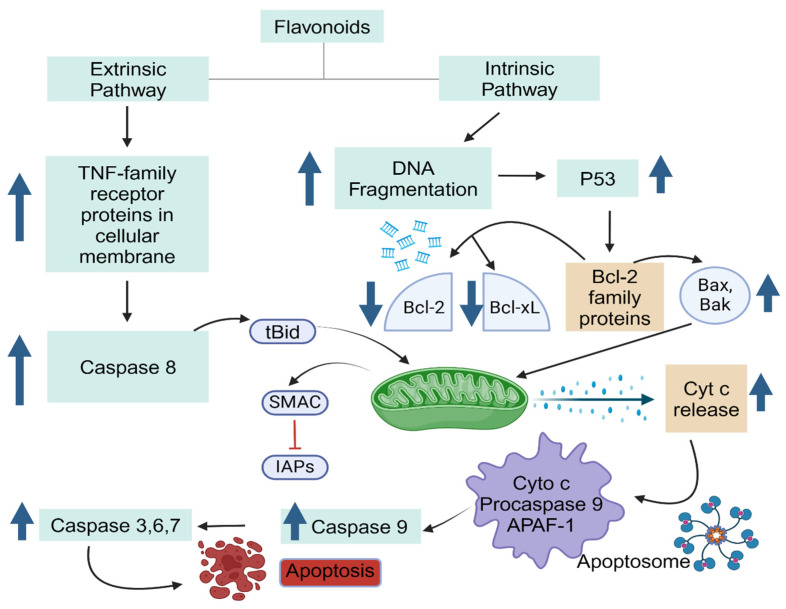
Flavonoid targets in the extrinsic and intrinsic apoptosis pathways: key molecules and signaling cascades.

Cancer cells often display resistance to apoptosis, a cellular process typically initiated by various signal transduction pathways and involves proapoptotic proteins such as caspases and the Bcl-2 family [131,132]. As shown in Figure 4, there are two primary signaling pathways of apoptosis: the extrinsic pathway, which is associated with the tumor necrosis factor (TNF) superfamily and is predominantly mediated by caspase 8; and the intrinsic pathway, also known as the mitochondrial pathway, which involves the Bcl-2 family proteins triggering the activation of caspases 9, 3, and 7 (Figure 4) [70]. In cancer cells, there is often an upregulation of oncogenic genes like c-Myc, promoting cellular proliferation and suppressing p53 function. Additionally, antiapoptotic proteins from the Bcl-2 family are activated, while proapoptotic proteins and caspases may be downregulated [133]. Flavonoids have the potential to intervene in apoptotic signaling cascades, thereby activating cell death pathways [75].

### 3.3. Anti-Hypertensive Effects of Flavonoids

Cardiovascular disease is the leading cause of death worldwide. Several key risk factors contribute to these conditions, including high blood pressure, aging, obesity, dyslipidemia, a sedentary lifestyle, smoking, stress, and poor lifestyle choices [134]. Nitric oxide released from endothelium plays a vital role in controlling vascular tone and blood pressure. Nitric oxide exerts its effects by triggering the cGMP-protein kinase G pathway in vascular smooth muscle cells. The activation of this pathway stimulates potassium channels, leading to membrane hyperpolarization and reduced intracellular calcium influx, which ultimately results in vasodilation. Protein kinase G achieves this effect by phosphorylating myosin light chains, thereby reducing the contractility of smooth muscles in blood vessels [135,136].

Flavones, a subgroup of flavonoids rich in luteolin, lower blood pressure by initiating and activating the cAMP/protein kinase A pathway. This pathway subsequently activates nitric oxide synthase, leading to an elevated concentration of endothelial nitric oxide. The resulting vasodilation is regulated by potassium and calcium channels [92,137]. The blood pressure-lowering effects of flavonols, such as kaempferol and quercetin, are achieved through modulation of the renin–angiotensin–aldosterone system, enhancement of the endothelial function, and regulation of smooth muscle contraction in blood vessels [138,139]. These mechanisms are attributed to their capability to activate nitric oxide-synthase 3, leading to increased plasma levels of nitric oxide. The enhancement of endothelial function is achieved by inhibiting the response of smooth muscle cells in blood vessels to endothelin-1 [140,141]. The dietary intake of quercetin varies across different countries. Estimated flavonoid consumption ranges from 50 to 800 mg per day, with quercetin making up approximately 75% of this intake. This variation largely depends on the consumption of fruits, vegetables, and tea. In vitro studies involving endothelial denudation have shown that the antihypertensive effects of quercetin and kaempferol rely on nitric oxide synthesized in the endothelium. This conclusion was drawn from the significant reduction in the vasodilator activity that was observed of these two flavonols when the endothelium was removed [142,143]. An in vivo study by Manach et al. [144] demonstrated that dietary flavonols, specifically quercetin and rutin, are absorbed and present in rat plasma as conjugated metabolites at concentrations around 115 µmol/L, and they also exhibit significant antioxidant properties, particularly in inhibiting LDL oxidation. Another study by Edwards et al. showed that quercetin administered for 4 weeks at a dose of 730 mg/day to individuals with prehypertension and stage 1 hypertension resulted in a reduction in blood pressure, but it did not affect the oxidative stress parameters in blood and urine [145].

In contrast to quercetin, naringenin, a member of the flavanone class, demonstrates vasodilatory effects even in the absence of the endothelium. This is due to its ability to activate potassium channels, especially those that are calcium-activated and voltage-dependent. Naringenin’s blood pressure-lowering effects are a result of membrane hyperpolarization and the relaxation of vascular smooth muscle, which are both influenced by calcium-activated potassium channels [146]. A study on hypertensive rats demonstrated that epicatechin’s antihypertensive mechanism involves decreasing superoxide production in the aorta and left ventricle, as well as in enhancing nitric oxide-synthase activity [147].

Soy isoflavones are structurally like human estrogen, which is believed to contribute to their beneficial role in preventing hypertension in menopausal women. Daidzein induces vasodilation through mechanisms similar to other flavonoids, but it also uniquely stimulates the production of prostaglandins [148]. Genistein’s antihypertensive effects are achieved by inhibiting the tyrosine kinase Pyk2, which regulates calcium ion channels and activates signaling pathways [149]. Additionally, genistein helps reduce pulmonary hypertension by decreasing smooth muscle hypertrophy in pulmonary arteries [150].

### 3.4. Anti-Microbial Effects of Flavonoids

Flavonoids demonstrate broad-spectrum antimicrobial activity against bacteria, fungi, and viruses [151]. They achieve this by disrupting microbial cell membranes, inhibiting nucleic acid synthesis, and interfering with microbial metabolism [152]. A recent study identified four compounds—pectolinaringenin (1), (±)-4′,5,7-trimethoxy flavanone (2), 5-hydroxy-3,7,4′-trimethoxyflavone (3), and 3,5,7-trihydroxy-4′-methoxyflavone (4)—from the South African weed *Chromolaena odorata*. Compounds 2 and 3 exhibited promising antimicrobial activity against *E. coli*, *S. aureus*, *K. pneumoniae*, *A. fumigatus*, and *C. neoformans*, with minimum inhibitory concentrations (MIC) between 0.016 and 0.125 mg/mL, which is comparable to gentamicin, ciprofloxacin, and amphotericin B. These compounds also demonstrated good anti-biofilm and metabolic inhibition activities but weak anti-adhesion effects. They showed low toxicity to human and animal cells, with selectivity indexes between 1 and 12.625, indicating higher toxicity to microbial strains. These findings suggest that Compounds 2 and 3 could be potential leads for developing prophylactic treatments and anti-infective drugs against urinary tract infection [153].

To comprehend the mechanisms through which dietary flavonoids exert their effects in the body, it is essential to identify the specific chemical forms of their various metabolites that are present in systemic circulation, as these are the physiologically active forms [154]. Dietary flavonoids are primarily found in their glycoside forms. However, in plasma, glycosides are rare due to the deglycosylation process that occurs in both the small and large intestines, which vary based on the type of sugar moiety [155,156,157]. In the small intestine, two enzymes act as β-glucosidases against flavonoid monoglucosides [158]. Lactase-phlorizin hydrolase (LPH), a brush border-associated enzyme, hydrolyzes lactose into glucose and galactose. Day et al. discovered that LPH also hydrolyzes quercetin 3-O-glucoside (Q3G), quercetin 4′-O-glucoside (Q4′G), and the monoglucosides of genistein and daidzein to produce corresponding aglycons in vitro [159]. The other enzyme, cytosolic β-glucosidase (CBG), located in enterocytes, shows broad specificity and hydrolyzes Q4′G, genistein 7-O-glucoside (genistin), and daidzein 7-O-glucoside (daidzin), but not quercetin 3,4′-O-diglucoside, Q3G, or quercetin 3-O-rhamnoglucoside (rutin), using cell-free extracts from the human intestine and liver [160]. Before CBG can hydrolyze these glucosides, they are taken up into the cell via the sodium-glucose co-transporter type 1 (SGLT1), as evidenced by studies using human Caco-2 cells and SGLT1-transfected rodent G6D3 cells [161]. In individuals with lactase deficiency, plasma levels of isoflavone metabolites are initially lower but eventually become like those in lactase-sufficient individuals, likely due to compensation by the intestinal microbiota. Isoflavone glucosides are primarily hydrolyzed by CBG in cell-free extracts of the human intestine and liver, with LPH playing a lesser role compared to rats [162]. Recently, calycosin-7-O-glucoside, a methylated isoflavone, was identified as a novel substrate for SGLT1 but not for LPH in rats [163]. Therefore, deglycosylation in the small intestine is crucial for enhancing the bioavailability of flavonoid monoglucosides. Nevertheless, the intestinal microbiota can often compensate for the absence of this process, ensuring sufficient hydrolysis and absorption of these compounds [164]. In recent years, numerous studies have highlighted the dual role of the gut microbiota in maintaining host health. Gut-resident bacteria produce various metabolites and bioproducts essential for protecting host and gut homeostasis. Conversely, during pathological dysbiosis, certain microbiota subpopulations can expand and produce high levels of toxins, leading to inflammation and tumorigenesis [165]. Flavonoids such as quercetin, epigallocatechin gallate (EGCG), and genistein have been demonstrated to alter the composition of the microbiota within the tumor microenvironment, resulting in changes in immune response and tumor growth [166]. A study by Maheswari et al. demonstrated that titanium dioxide nanoparticles modified with extracts from *Plectranthus amboinicus* (*Karpooravalli*), *Phyllanthus niruri* (*Keezhanelli*), and *Euphorbia hirta* (*Amman Pacharisi*) were synthesized using the hydrothermal method. X-ray diffraction revealed the anatase nature of the samples, and TEM analysis showed an increase in particle size for the bio-modified samples. These nanoparticles exhibited significant antibacterial activity against both Gram-negative (*Escherichia coli*, *Klebsiella pneumoniae*, and *Pseudomonas aeruginosa*) and Gram-positive bacteria (*Staphylococcus aureus* and *Streptococcus* mutans), with *Plectranthus amboinicus*-modified titanium dioxide nanoparticles showing the best results [167].

## 4. Flavonoid-Mediated Nanoparticles and Their Efficacy

South Africa is home to approximately 10% of the world’s plant species, with over 3000 species recognized for their significant medicinal properties [23]. Medicinal plants have a longstanding role in traditional medicine, with over 80% of the South African population depending on them for primary health care [168]. Medicinal plants provide diverse therapeutic benefits due to their rich array of secondary metabolites, including flavonoids, alkaloids, phenolics, terpenoids, tannins, glycosides, quinones, steroids, and saponins [169]. Flavonoids are commonly formulated into various food supplements to leverage their health benefits, enhancing both their bioavailability and efficacy for consumers. Formulating flavonoids into these supplements can improve their bioavailability, which is often low due to poor solubility and rapid metabolism [19]. Techniques such as nanoparticle encapsulation, liposomal delivery, and complexation with cyclodextrins are used to increase the absorption and stability of flavonoids [170]. Several phytochemical and pharmacological studies of these plants and their derivatives have shown impressive in vitro activity but less in vivo efficacy. To enhance their effectiveness, a growing body of literature is suggesting combining traditional medicinal plants with nanotechnology [171].

Despite this rich biodiversity and the potential advantages of plant-mediated metallic nanoparticles (MNPs), the use of South African medicinal plants for MNP synthesis remains largely underexplored [172]. Recently, several indigenous South African plants, including *Salvia africana-lutea*, *Sutherlandia frutescens*, *Galenia africana*, *Catharanthus roseus*, *Hypoxis hemerocallidea*, *Cotyledon orbiculata*, and *Aspalathus linearis*, have been employed in the synthesis of MNPs [173]. These nanoparticles, with sizes ranging from 5 to 50 nm, have demonstrated significantly higher antibacterial activities compared to their corresponding plant extracts [174,175]. Given the substantial potential of plants as alternative sources of reducing agents with enhanced bioactivity, ongoing research into the rich medicinal plant reserves of South Africa is crucial [176]. Nanoparticles can exploit the enhanced permeability and retention (EPR) effect, which is a phenomenon where nanoparticles tend to accumulate in tumor tissue much more than in normal tissues due to the leaky vasculature and poor lymphatic drainage in tumors. Quercetin nanoparticles have shown higher accumulation in tumor sites compared to free quercetin [177]. Nanoparticles can be engineered with surface modifications to target specific tissues or cells, enhancing their distribution to desired sites within the body. Flavonoid nanoparticles functionalized with antibodies or ligands can target cancer cells specifically, improving therapeutic outcomes [178]. They can also increase the solubility of flavonoids that are poorly soluble in water, leading to better absorption in the gastrointestinal tract. Nanoparticles can shield flavonoids from the degradation caused by enzymes and acidic conditions in the gastrointestinal tract, enhancing their stability and bioavailability. Encapsulating flavonoids in polymeric nanoparticles has proven effective in preventing early degradation and boosting their therapeutic efficacy [179]. Nanoparticles not only protect flavonoids from degradation in the gastrointestinal tract, but also enable controlled release, maintaining therapeutic levels in the bloodstream over extended periods. For example, the sustained release of EGCG from nanoparticles has demonstrated enhanced bioavailability and prolonged antioxidant effects [180].

Green nanobiotechnology involves the creation of nanoparticles or nanomaterials using biological methods, harnessing the abilities of microorganisms, plants, viruses, or their components, like proteins and lipids, aided by biotechnological techniques [181]. Plants have served as natural remedies for various physiological disorders in traditional Eastern medicine, notably in Indian and Chinese practices, since ancient times. There is existing literature on the ‘Green’ synthesis of nanoparticles such as copper (Cu), gold (Au), nickel (Ni), platinum (Pt), titanium (Ti), selenium (Se), silver (Ag), and zinc (Zi) using plant resources [182]. These plant-based metal nanoparticles have demonstrated remarkable antimicrobial, anticancer, antidiabetic, anti-inflammatory, antioxidant, and immunomodulatory activities, as reported in previous studies [183,184]. Previous research has consistently indicated that the reduction and stabilization of metal ions during synthesis are attributed to the presence of phytochemicals in plant materials. These phytochemicals encompass alkaloids, flavonoids, phenols, terpenoids, alcohols, sugars, and proteins [185,186]. While synthesizing MNPs using a singular active substance from plant extracts can aid in nanoparticle purification, additional research is necessary to explore the biomedical applications of such MNPs for treating specific diseases. Currently, there is limited literature available on the use of a single substance from plant extracts for MNP synthesis. Recent findings have emphasized the significant role of flavonoids, which are widely present in plant extracts, in the bio reduction of metal ions leading to nanoparticle formation [187,188]. A recent innovative study found that *Karpooravalli–Keezhanelli*-modified *titanium dioxide nanoparticles* demonstrated minimal cell viability at a concentration of 50 µg mL^−1^, indicating excellent anticancer activity. Subsequent MTT assays on normal L929 cells showed these nanoparticles to be safer and less toxic. Therefore, *Karpooravalli–Keezhanelli*-modified titanium dioxide nanoparticles have potential as future anticancer drugs [167]. Table 3 provides a comprehensive overview of various nanoparticles (NPs) synthesized using different flavonoids, each exhibiting unique properties and potential applications. Quercetin-based silver and selenium nanoparticles (Ag-SeNPs) have a size range of 30–35 nm [189]. The selection of the nanocarrier is based on the structure of quercetin, specifically its 2-(3,4-dihydroxyphenyl)-3,5,7-trihydroxy-4H-chromen-4-one configuration [29]. Quercetin can scavenge free radicals that are generated by metals. Mittal et al. proved quercetin is mainly responsible for the reduction and stabilization of metal ions. Interestingly, the aforementioned study confirmed quercetin-mediated bimetallic Ag-SeNPs possess higher therapeutic efficacy in terms of antioxidant, antimicrobial, and anticancer activities [189]. Kaempferol, a flavanol widely found in various plant sources, has been investigated for its involvement in the synthesis of gold nanoparticles (AuNPs). In a study conducted by Raghavan et al. [190], it was revealed that kaempferol plays a crucial role in the formation of AuNPs. Furthermore, the resulting nanoparticles exhibit considerable potential in the realm of cancer treatment, particularly showing promise in combating human breast cancer [190]. Apiin-coated AuNPs with a size of 21 nm display notable anticancer activity [191]. Proanthocyanidin-coated AuNPs, sized between 17 and 29 nm, exhibit efficient cardio-protective potential along with good biocompatibility [192]. Luteolin-conjugated silver nanoparticles (AgNPs) at size of 13 nm exhibit significant antimicrobial activity against Bacillus subtilis [193]. Baicalein, a major bioactive flavonoid, demonstrates significant synergistic effects when combined with tetracycline and β-lactams in the treatment of methicillin-resistant *Staphylococcus aureus* (MRSA). Additionally, it has been noted for its ability to mitigate quorum sensing-controlled virulence factors, particularly biofilm formation, in *Pseudomonas aeruginosa* [194]. In a study by Rajkumari et al. the synthesis of AuNPs mediated by baicalein was reported. Baicalein-based AuNPs at a size of 39 nm demonstrate antibiofilm activity against *Pseudomonas aeruginosa* [195]. Flavonoids from *Dalbergia spinosa*, when used in the synthesis of silver nanoparticles (AgNPs) at a size of 18 nm, exhibit both anti-inflammatory and antibacterial activities against *Escherichia coli*, *P. aeruginosa*, *Staphylococcus aureus*, and *B. subtilis* [196]. These findings highlight the versatility and diverse applications of bioactive compounds in nanoparticle synthesis. The nanoparticles show potential in areas such as cancer treatment, antimicrobial applications, and anti-inflammatory responses. The varied sizes and compositions of the nanoparticles also contribute to their unique functionalities, demonstrating the broad spectrum of possibilities in the field of nanomedicine and nanotechnology.

Numerous studies have explored the interactions between flavonoids and metal nanoparticles, particularly those involving Ag and Au, as well as the oxides of Fe, Zn, and Ti, resulting in materials with intriguing physicochemical and biological properties. Silver nanoparticles have shown promising antioxidant, antibacterial, antifungal, antiparasitic, antiviral, and anticancer properties. To investigate potential medical applications, silver nanoparticles have been functionalized with various flavonoids such as apigenin, catechin, EGCG, kaempferol, myricetin, 4′,7-dihydroxyflavone, dihydromyricetin, hesperidin, and quercetin. Moreover, silver@quercetin nanoparticles have been studied as biocompatible and photostable aggregation-induced emission luminogens for the in situ and real-time monitoring of biomolecules and biological processes. In biological studies, silver nanoparticles combined with quercetin have been examined for their potential anti-inflammatory effects. Additionally, silver nanoparticles loaded with isoorientin have been investigated for their potential toxicity and their activity on enzymes related to type II diabetes and obesity [197]. In a 2021 study, Kollur et al. investigated the anticancer properties of luteolin-functionalized zinc oxide nanoparticles (L-ZnONPs). These nanoparticles were synthesized by combining an aqueous solution of Zn(OAc)_2_ with luteolin, followed by filtration of the resulting white precipitate, ethanol washing to eliminate impurities, and calcination of the final product [198]. Results suggest that the nanohybrid induces intrinsic cellular mechanisms leading to apoptosis and halts the cell cycle. Moreover, by downregulating MMP-2 and VE-cadherin expression, the nanoparticles suppress the migration and invasion of A549 cells [198]. In another investigation, Salaheldin et al. explored the potential application of three novel nano formulations of (-)-epigallocatechin-3-gallate (EGCG) as natural chemo preventive agents against DNA damage induced by ultraviolet beam (UVB) radiation in keratinocytes [199].

Gold nanoparticles (AuNPs), much like silver nanoparticles (AgNPs), have garnered significant attention since their discovery. This is primarily attributed to their straightforward synthesis, easy surface modification, and their broad spectrum of potential applications, particularly in the field of medical sciences [200]. Gold nanoparticles are frequently functionalized with flavonoid compounds to enhance their properties and expand their range of applications [201]. Gold nanorods integrated with metal–phenolic networks, which include EGCG, procyanidins (OPC), and tannic acid (TA), have demonstrated significantly accelerated wound healing and strong bactericidal effects when used with NIR irradiation. Similarly, quercetin-conjugated gold nanoparticles have shown both antibacterial and antioxidant activities. Additionally, gold nanoparticles coated with CHY, kaempferol, and quercetin have exhibited enhanced antibacterial efficacy against Gram-negative bacteria [202,203]. In a study by Sivakumar et al. [204], suggested silver nanoparticles (AgNPs) were phytosynthesized using leaf extract from *Phyllanthus urinaria L*. They proposed that polyols like flavones and catechins play a role in reducing silver ions to form AgNPs, and they are supported by the disappearance of the C-O band at 1226 cm^−1^ in IR spectra. The nanoparticles range in shape from cuboidal to orthorhombic, with sizes varying from 15 nm to 80 nm. Antimicrobial tests against *Escherichia coli*, *Salmonella typhi*, *Vibrio cholera*, *Pseudomonas aeruginosa*, and *Proteus mirabilis* showed strong bactericidal activity across all tested concentrations (100 to 400 µg/mL). Maximum inhibition zones of 18 mm were observed against *V. cholera* at 400 µg/mL, while the lowest activity was 8 mm against *P. mirabilis* at 100 µg/mL. Further research is needed to determine the minimum inhibitory concentration (MIC) values for comprehensive efficacy assessment [204].

Flavonoid-modified metal nanoparticles exhibit their usefulness starting from the initial synthesis stage to advanced investigations in medical and pharmaceutical domains. Understanding various methods for synthesizing flavonoid–metal nanoparticle conjugates and hybrids, along with their characterization, biological attributes, and medical uses, offers insights into numerous possibilities. Numerous research outcomes have indicated that combining flavonoids with metal nanoparticles enhances their individual characteristics and potential applications.

## 5. Conclusions and Suggestions

In conclusion, this review highlights the significant potential of flavonoid-mediated nanoparticles, especially those derived from South African plant species, as promising therapeutic agents owing to their potent antioxidant and anti-inflammatory properties. The diverse flora in South Africa provide a rich source of these compounds, opening avenues for future research and development, particularly in the context of nanomedicine. Exploring the unique aspects of flavonoid-mediated nanoparticles, such as their size, structure, and interactions, presents novel dimensions for enhancing therapeutic efficacy.

Further investigations into the distinctive mechanisms of action, bioavailability, and potential synergistic effects of flavonoid-loaded nanoparticles across various plant species offer exciting prospects for the development of innovative therapies. Understanding these nanoparticles behavior and leveraging traditional knowledge with modern scientific approaches are crucial for unlocking their full potential in treating a spectrum of diseases.

## Figures and Tables

**Table 1:** Diversity of South African plant species and their flavonoid-rich compositions.

Plant Species	Flavonoids	Traditional Uses	References
*Rooibos* (*Aspalathus linearis*)	Flavonols like quercetin and aspalathin	A traditional herbal infusion that is consumed for its refreshing taste and potential health benefits	[30,31]
*Cyclopia intermedia* (Honey bush)	Various flavonoids	Soothing coughs and easing respiratory issues like tuberculosis, pneumonia, and catarrh	[33]
*Hoodia gordonii*	Quercetin, kaempferol	Appetite-suppressing properties	[34,35]
*Cycads* (Various species)	Various flavonoids among diverse phytochemical constituents	Consumption as a starchy food source, raising conservation concerns	[37]
*Pelargonium sidoides* (*Umckaloabo*)	Quercetin derivatives	Treating respiratory infections	[38]
*Sceletium tortuosum*	Mesembrine alkaloids (distinctive flavonoid type)	Mood enhancement and stress reduction	[40]

**Table 2:** Anticancer effects and the mechanism of action of various flavonoids.

Flavonoids	Cancer Type	Cell Line	Effect	Mechanism	References
Genistein	Breast cancer	MDA-MB-231 and MCF-7	G2/M phase arrest, Apoptosis	ROS dependency	[92]
Daidzein	Breast cancer	MCF-7	Apoptosis induction	ROS generation	[93]
Hesperetin	Gall bladder, esophageal, hepatocellular, and breast cancer	MCF-7	Apoptosis trigger	Mitochondrial pathway, ROS production	[94,95,96,97]
Naringenin	Choriocarcinoma, epidermoid carcinoma, and prostate cancer	JAR, JEG 3 A431, PC3 and LNCaP	Anticancer effects	ROS generation, apoptotic pathways	[98,99,100]
Cocoa Catechins/Procyanidins	Ovarian cancer	OAW42 and OVCAR3	Apoptosis	DNA damage, apoptotic changes	[103]
Cocoa Catechins/Procyanidins	Adenocarcinoma	Caco2	Oxidative Stress Protection	Reduced ROS production	[109]
Cocoa Polyphenolic Extract	Liver cancer	HepG2	ERK1/2 pathway activation	Increased antioxidant enzyme activity	[108]
Quercetin	Liver cancer	HepG2	Chemo preventive properties	Reduced proliferation, decreased ROS levels	[111]
Quercetin	Gastric adenocarcinoma and breast cancer	AGS and MCF-7	Apoptosis induction	Increased ROS production	[112]
Kaempferol	Bladder cancer	EJ	Growth inhibition	Apoptosis, S phase arrest, ROS modulation	[113]
Kaempferol	Colorectal cancer	HCT116, HCT15, and SW480	Apoptosis activation	Caspase activation, ROS generation	[114]
Kaempferol	Hepatocellular carcinoma	HepG2	Cytotoxic effects	Mitochondrial targeting, ROS mediation	[115]
Apigenin	Ovarian cancer	A2780, OVCAR-3, and SKOV-3	Apoptosis promotion	ROS signaling alteration	[116]
Apigenin	Cervical cancer	HeLa, SiHa, CaSki, and C33A	Apoptosis activation	ROS generation, mitochondrial pathway	[117]
Chrysin	Choriocarcinoma, bladder cancer, and ovarian cancer	JAR, JEG3, ES2 and OV90	Induction of death	Increased ROS, lipid peroxidation	[118,119]
Cyanidin	Prostate cancer	DU145 and LnCap	Induced cell death	ROS modulation	[121]
Cyanidin and Delphinidin	Colorectal cancer	LoVo and LoVo/ADR	Cytotoxic effects	ROS accumulation	[122]

**Table 3:** Flavonoid-mediated nanomaterials and their sizes with applications in biomedicine.

Flavonoids	Nanomaterials	Size (nm)	Biomedical Applications	References
Quercetin	Ag-SeNPs	30–35 nm	Exhibit antioxidant, antimicrobial, and anticancer activities.	[189]
Proanthocyanidin	AuNPs	17–29 nm	Efficient cardio-protective potential with good biocompatibility	[192]
Luteolin	AgNPs	13 nm	Antimicrobial activity against *B. subtilus*	[193]
Kaempferol	AuNPs	16.5 nm	Anticancer activity against human breast cancer.	[190]
Apiin	AuNPs	21 nm	Anticancer activity	[191]
Baicalein	AuNPs	39 nm	Antibiofilm activity against *P. aeruginosa*	[195]
Flavonoids (*Dalbergia spinosa*)	AgNPs	18 nm	Anti-inflammatory and antibacterial (*E. coli*, *P. aeruginosa*, *S. aureus*, and *B. subtilis)* activities	[196]
